# Transfer-Printed Cuprous Iodide (CuI) Hole Transporting Layer for Low Temperature Processed Perovskite Solar Cells

**DOI:** 10.3390/nano12091467

**Published:** 2022-04-26

**Authors:** Ravi P. Srivastava, Hyun-Suh Jung, Dahl-Young Khang

**Affiliations:** Department of Materials Science and Engineering, Yonsei University, Seoul 03722, Korea; ravimme.iitk@gmail.com (R.P.S.); li5245@naver.com (H.-S.J.)

**Keywords:** perovskite solar cells, hole-transporting layer, cuprous iodide, transfer printing, ambient environment processing

## Abstract

Perovskite solar cells (PSCs) have achieved significantly high power-conversion efficiency within a short time. Most of the devices, including those with the highest efficiency, are based on a n–i–p structure utilizing a (doped) spiro-OMeTAD hole transport layer (HTL), which is an expensive material. Furthermore, doping has its own challenges affecting the processing and performance of the devices. Therefore, the need for low-cost, dopant-free hole transport materials is an urgent and critical issue for the commercialization of PSCs. In this study, n–i–p structure PSCs were fabricated in an ambient environment with cuprous iodide (CuI) HTL, employing a novel transfer-printing technique, in order to avoid the harmful interaction between the perovskite surface and the solvents of CuI. Moreover, in fabricated PSCs, the SnO_2_ electron transport layer (ETL) has been incorporated to reduce the processing temperature, as previously reported (n–i–p) devices with CuI HTL are based on TiO_2_, which is a high-temperature processed ETL. PSCs fabricated at 80 °C transfer-printing temperature with 20 nm iodized copper, under 1 sun illumination showed a promising efficiency of 8.3%, (J_SC_ and FF; 19.3 A/cm^2^ and 53.8%), which is comparable with undoped spiro-OMeTAD PSCs and is the highest among the ambient-environment-fabricated PSCs utilizing CuI HTL.

## 1. Introduction

**Figure 1:**
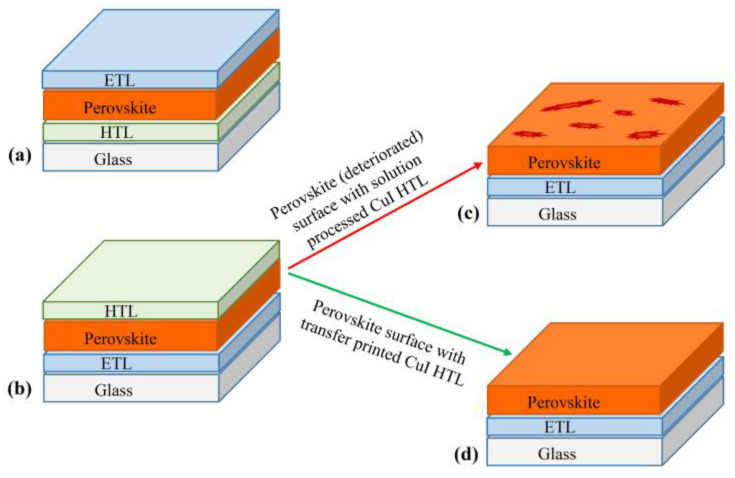
(**a**) p–i–n and (**b**) n–i–p structures for the perovskite solar cells. In a n–i–p structure, a perovskite surface with (**c**) solution-processed (deterioration of perovskite surface due to harmful interaction between solvents of CuI and perovskite) and (**d**) transfer-printed CuI HTLs.

Organometal halide perovskite materials with numerous advantages such as an appropriate band gap, a high absorption coefficient (1.5 × 10^4^ cm^−1^ at 550 nm), a broad range of light absorption (including the visible to near-infrared spectra) with a long carrier diffusion length (~1000 nm) and high defect-tolerance are very promising for next-generation photovoltaics [1,2,3,4,5,6,7]. The PSCs utilizing these materials can be manufactured in two different structures, depending on the order of deposition of the electron transport layer (ETL) and the hole transport layer (HTL). The so-called structures are the “standard (n–i–p) structure” (negative–intrinsic–positive) and the “inverted (p–i–n) structure” (positive–intrinsic–negative) (Figure 1a,b). At present, both of the structures have achieved high power-conversion efficiencies (PCEs) above 20–22% [8,9,10], but n–i–p-structure PSCs have showed the record efficiency for single-junction cells [9,11,12]. In comparison, for p–i–n structure PSCs, using similar perovskites, typical efficiencies remain significantly lower [13]. This difference may be attributed to lower open-circuit voltage (V_OC_) for p–i–n-structure PSCs, due to higher non-radiative recombination resulting from the non-suitable doping of the perovskite near its n-type interface [14]. In both structures, the role of HTL is very crucial, as it helps in the efficient charge extraction of photogenerated holes, inhibits direct contact between the perovskite and the electrode and suppresses the charge recombination losses.

At the present time, spiro-OMeTAD is the most commonly used HTL in n–i–p-structured PSCs. However, the production of spiro-OMeTAD requires an ultra-low temperature (−78 °C) and multiple synthesis steps, making the process complicated and highly expensive (~300 $/g) [15]. Additionally, the use of spiro-OMeTAD requires doping with lithium bis- (trifluoromethylsulfonyl)imide (Li-TFSI) and 4-tert-butylpyridine (4-tBP), which affects the performance (stability) of the devices [16]. Therefore, the development of low-cost dopant-free HTLs is extremely important for the progress and commercialization of the PSC industry. In this regard, considerable development has occurred over the past few years, and different dopant-free hole-transport materials have also been explored for the planar n–i–p-structured PSCs (Table S1, Supplementary Materials). For the development and growth of the PSCs industry, besides power-conversion efficiency, stability (chemical and thermal), electrical properties (conductivity and hole mobility), method of synthesis (for precursor materials and devices) and cost (raw material and processing) are major concerns [17,18]. Most of the reported organic HTMs have been synthesized by complicated multistep methods requiring controlled (argon/nitrogen) environments for a long time, in addition to costly purification techniques. Moreover, various chemical solvents and precursors used in the synthesis are not eco-/user-friendly [19,20,21,22,23,24,25,26,27,28,29,30]. Considering these points, inorganic materials, such as CuSCN, CuI and NiO, were found to be potential candidates for replacing expensive hole-transport materials [31,32,33,34,35]. Among these, low-cost cuprous iodide (CuI), which possesses several good characteristics, such as high hole-mobility, high transparency and good chemical stability, is a potential candidate that can be synthesized in an ambient environment with very simple and less time-consuming methods compared to other HTMs (Table S1) [36,37]. Undoubtedly, CuI has vast advantages over spiro-OMeTAD, but its deposition in n–i–p-structured PSCs is still a challenge, due to the harmful interaction of its solvent with the perovskite surface (Figure 1c). Although different methods have been reported for the deposition of the CuI layer, such as spin-coating, spray-coating, thermal evaporation and powder-pressing [38,39,40,41,42], but most are chemical-deposition approaches, using a CuI solution dissolved in acetonitrile or a di-n-propyl sulfide/chlorobenzene mixture [32,39,43]. Acetonitrile is quite destructive to the perovskite layer; therefore, attempts have been made to replace it with a di-n-propylsulfide/chlorobenzene mixture. However, due to the fast reaction of this mixture with perovskite, the application of the CuI solution needs to be very quick to ensure that di-n-propyl sulfide does not affect the perovskite layer. These issues restrict the use of CuI HTL in the n–i–p-structured PSCs; hence, a suitable deposition method that does not have any side effect on the perovskite layer is the main challenge for the use of inorganic CuI HTL in PSCs (Figure 1d). In addition, most of the previously fabricated devices with CuI HTL are based on the TiO_2_ ETL, which requires high-temperature processing, limiting the fabrication process and restricting the choice of substrates for the devices.

Motivated by the abovementioned points, i.e., higher cost, complicated synthesis routes, corrosion and damage of perovskite surface with the solvents of CuI and the higher processing temperature of TiO_2_ ETL, the present study was carried out to investigate the use of a low-cost hole-transporting material (CuI) in low-temperature-processed perovskite solar cells. A novel solvent-free transfer-printing method has been employed for the CuI hole-transport-layer deposition in the n–i–p-structured PSCs using low-temperature-processed SnO_2_ ETL. The perovskite solar cells were fabricated in a fully ambient environment. Under 1 sun (100 mW/cm^2^) illumination, optimized devices with transfer-printed CuI HTL showed a promising efficiency of 8.3%, (short circuit current density and FF; 19.3 mA/cm^2^ and 53.8%), which is the highest among the open-environment-fabricated PSCs with CuI HTLs. For a performance comparison, devices with (doped and undoped) spiro-OMeTAD were also fabricated. The devices with CuI HTL showed a PCE comparable to those fabricated with undoped spiro-OMeTAD.

## 2. Materials and Methods

### 2.1. Materials

SnO_2_ -ETL was fabricated with the SnO_2_ colloid precursor (tin (IV) oxide, 15% in H_2_O colloidal dispersion) purchased from Alfa Aesar. For the fabrication of the PSCs, formamidinium iodide (>99.99%) and methylammonium bromide (>99.99%) were purchased from Greatcell Solar. Lead bromide (PbBr_2_, >98%) and lead (II) iodide (PbI_2_, 99.99%) were obtained from TCI chemicals. Bis(trifluoromethylsulfonyl)amine lithium salt (Li-TFSI, 99.95%), methylammonium chloride (MACl, >98%), dimethyl sulfoxide (DMSO, 99.9% anhydrous), dimethylformamide (DMF, 99.8%), hexane (>98%), ethyl acetate (>99%), 4-tertbutylpyridine (t-BP, 98%), iodine (≥99.99%) and copper (99.999%) were obtained from Sigma-Aldrich and used without any purification. 2,2′,7,7′-Tetrakis[N,N-di(4-methoxyphenyl)amino]-9,9′-spirobifluorene, (Spiro-OMeTAD, >99%) for the deposition of the hole-transport layer (HTL) was obtained from Lumtech. Polydimethylsiloxane (PDMS) (Sylgard 184, Dow) was used for the PDMS stamp fabrication.

### 2.2. Fabrication of PDMS and Copper Iodide Stamps

For PDMS stamp fabrication, a curing agent and a base resin in a 1:10 ratio in weight were mixed and poured into a Petri dish. The mixture was cured at 80 °C for 2 h, afterwards removing trapped air bubbles in a vacuum desiccator for 1 h. Finally, the PDMS stamp was gently peeled off and cut into appropriate sizes and fixed on the glass slides. These glass/PDMS substrates were placed inside the e-beam evaporator for the copper deposition. Copper films with different thicknesses (20–30 nm) were deposited on these (glass/PDMS) substrates. For the iodization of glass/PDMS/Cu stamps, a small amount of iodine was kept inside a Petri dish (Figure S1 Supplementary Materials), and iodization was performed at different temperatures, ranging from 10–30 °C.

### 2.3. Fabrication of PSCs

The details of the fabrication of perovskite solar cells can be found in our previous report [44]. In brief, a hundred microliters of a diluted (2.5%) SnO_2_ solution was spin-coated at 4000 rpm for 35 s on the cleaned ITO substrates. The spin-coated films were annealed at 150 °C for 2 min, and subsequently, another layer of SnO_2_ was deposited with the same parameters. Finally, the films were annealed at 150 °C for 45 min on a hot plate in an open environment. The perovskite solution (PbI_2_ (530.0 mg), PbBr_2_ (75.0 mg), FAI (189.0 mg), MABr (18.5 mg) and MACl (4.5 mg) in 1 mL DMF/DMSO (3:1, *v*/*v*)) was spin-coated at 1000 rpm for 10 s with a ramping rate of 500 rpm/s, followed by 4000 rpm for 30 s with a ramping rate of 2000 rpm/s. Then, 100 μL anti-solvent (7:3 ethyl acetate and hexane) was dripped onto the spinning substrate 20 s prior to the end of the second step of spin-coating. Spin-coated perovskite layers were immediately annealed at 150 °C for 20 min on a hotplate in an ambient environment.

### 2.4. Fabrication of Hole-Transporting Layers

After the cooling of the perovskite layers to room temperature, a spiro-OMeTAD solution, consisting of 75 mg spiro-OMeTAD in one ml of chlorobenzene (for undoped spiro-OMeTAD HTLs), was spin-coated on perovskite films. For doped spiro-OMeTAD HTLs, a spiro-OMeTAD solution, consisting of 75 mg spiro-OMeTAD, 21 mL t-BP and 19 mL of LiTFSI solution (520 mg LiTSFI in one ml acetonitrile) in one ml of chlorobenzene, was spin-coated on perovskite films. In order to avoid the degradation of perovskite films due to the spiro-OMeTAD solution and to minimize the exposure of spiro-OMeTAD to the ambient atmosphere, the dynamic spin-coating method has been utilized. The spiro-OMeTAD solution was dripped on the rotating substrate 15 s before the completion of the program. For the CuI HTL-based devices the fabricated CuI stamps (Section 2.2) have been used for the transfer printing (details in Section 3). Finally, 80 nm gold-top electrodes were deposited with the e-beam evaporator on hole-transporting layers.

### 2.5. Characterizations

The crystal structures of the e-beam evaporated copper, iodized copper and perovskite films were determined by X-ray diffraction (XRD, Rigaku Smartlab) analysis using CuK_α_ radiation (1.54 Å) at room temperature. The morphology of the films was examined using a scanning electron microscope (S-5000, Hitachi). The cross-sectional images of the fabricated PSCs were obtained with a field emission scanning electron microscope (JEOL (JSM-7100F)). The steady-state photoluminescence spectra (SSPL) of the samples were obtained using a PicoQuant Fluo Time 300 with 510 nm laser excitation. The J–V curves of the fabricated devices were collected using a solar simulator (Newport AAA solar) equipped with a 150 W xenon lamp and a Keithley 2400 source meter under AM 1.5 simulated sunlight intensity adjusted to 1 sun illumination (100 mW/cm^2^). All measurements were performed by masking the cells with a metal aperture to define an active area of 0.04 cm^2^. Intensity calibration was performed using an NREL-calibrated silicon solar cell. Dark (J–V) for the fabricated PSCs were obtained under dark conditions.

## 3. Results and Discussion

**Figure 2:**
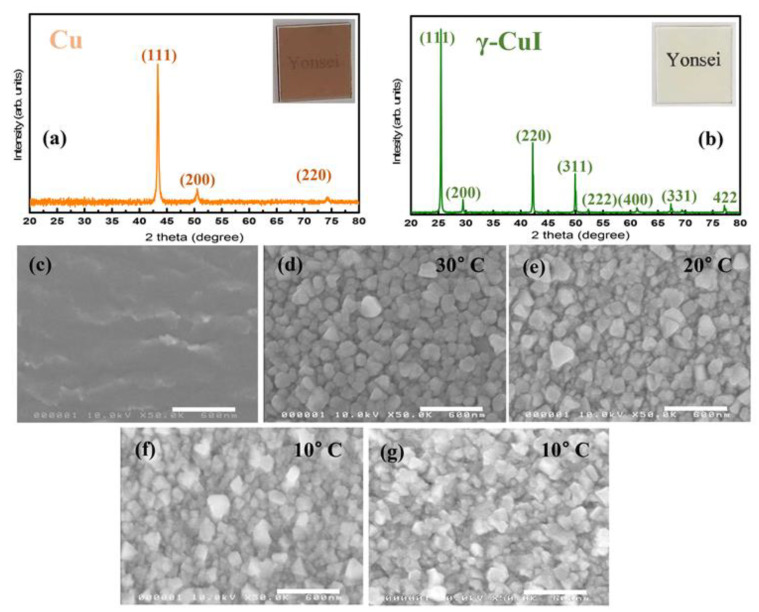
(**a**,**b**) XRD of Cu and iodized films on the glass substrate (inset shows the e-beam evaporated copper and iodized films, respectively). Morphology of (**c**) e-beam evaporated copper film. (**d**–**f**) Iodized films obtained after iodization of 20 nm copper films at different iodization temperatures; (**d**) 30 °C (**e**) 20 °C and (**f**) 10 °C. (**g**) Iodized films obtained at 10 °C from 30 nm thickness of copper film on glass/PDMS. Scale bars are 600 nm in (**c**–**g**).

Figure 2c–g shows SEM images of the Cu and iodized (CuI) films on the glass/PDMS substrate, obtained under different conditions. The copper films (Figure 2c) are smooth and uniform with small grains. Figure 2d shows the morphology of CuI films obtained at an iodization temperature (T_Iod_) of 30 °C. The films are loosely packed with lots of pin holes and openings between the grains. As morphologies of the electron/hole transporting layers are very crucial to device performance, the morphology of the iodized films was controlled with T_Iod_. The morphologies of the CuI films obtained at different T_Iod_ are shown in Figure 2d–f. From the figure it can be seen that, as T_Iod_ has decreased (30 to 10 °C), the average grain size of CuI films also decreased. The CuI film obtained at T_Iod_ of 10 °C is more compact and continuous, having negligible pin holes, while the films obtained at higher T_Iod_ show a large grain size and more pin holes, due to the rapid iodination process occurring at a relatively high temperature, eventually generating the pin holes on the film surface [45].

The transformation of Cu into CuI was examined with X-ray diffraction measurements (Figure 2a,b). X-ray diffraction for the e-beam evaporated film (Figure 2a) shows three peaks at 2*θ* values of 43.4°, 50.5° and 74.2° corresponding to (111), (200) and (220) planes of copper (JCPDS Card No. 04-0836) [46]. After iodization, the film exhibits major diffraction peaks at 2θ values of 25.4°, 29.4°, 42.1°, 49.8°, 67.3° and 77.1°, corresponding to (111), (200), (220), (311), (331) and (422) planes, respectively (Figure 2b). These peak values are in good agreement with the γ-CuI (JCPDS Card No. 06-0246) [47]. The relatively higher intensity of the peak at 25.4° in the iodized film indicates the preferred orientation of γ-CuI films along (111). In iodized films, all of the diffraction peaks are assigned to γ-CuI without Cu, α-CuI, β-CuI and/or any other impurity phases. In addition, the copper film with 30 nm thickness was also iodized under the same conditions to check the performance of PSCs with different thicknesses of CuI. The morphology of the CuI films obtained from the iodization of 30 nm of copper is shown in Figure 2g. The film is compact and shows similar morphology to those obtained with 20 nm thickness of Cu. The XRD and SEM morphology of fabricated perovskite films is shown in Figure S2 (Supplementary Materials). The XRD pattern (Figure S2a) confirms the perovskite structure of fabricated films with the presence of PbI_2_. The synthesized perovskite films are compact and uniform, with an average grain size of ~ 295 nm (Figure S2b). Based on the results of CuI films morphologies, devices were fabricated with the CuI obtained from the iodization of 20 and 30 nm copper films.

**Figure 3:**
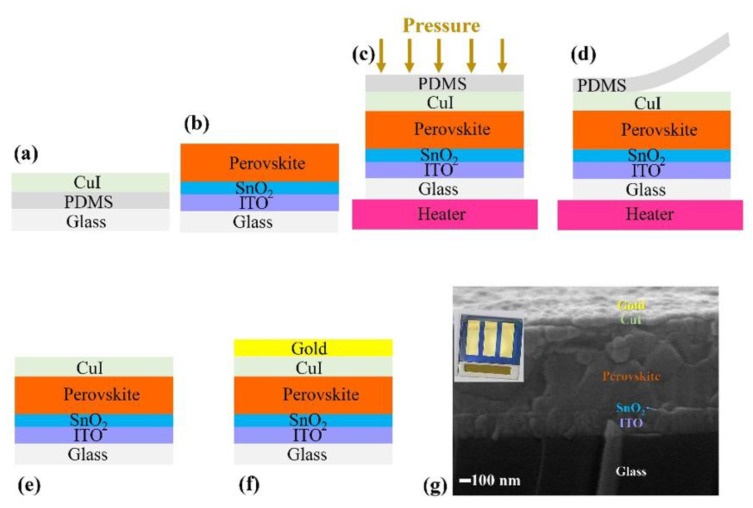
Schematic of device fabrication with transfer-printing of CuI. (**a**) Glass/PDMS/CuI stamp, (**b**) glass/ITO/SnO_2_/perovskite film, (**c**) CuI stamp and perovskite during transfer-printing, (**d**) PDMS detachment after transfer-printing, (**e**) glass/ITO/SnO_2_/perovskite/(transfer-printed) CuI, (**f**) glass/ITO/SnO_2_/perovskite/CuI/gold. (**g**) Cross-sectional image of PSC (inset laboratory-fabricated device) with transfer-printed CuI HTL.

A schematic diagram showing the fabrication of PSCs with transfer printing of CuI is presented in Figure 3. Figure 3a,b shows a glass/PDMS/CuI stamp (detailed information on stamp fabrication is described in Supplementary Materials Figure S1) and glass/ITO/SnO_2_/perovskite films, respectively. As discussed earlier, low-temperature iodization produced more compact CuI films; therefore, low-temperature-iodized stamps were placed on the top surface of perovskite films and pressured at transfer-printing pressure (P_TP_) of 4 bar at different transfer-printing temperatures (T_TP_) for 20 min of transfer-printing time (t_TP_). Figure 3c shows the glass/PDMS/CuI stamp and glass/ITO/SnO_2_/perovskite film during the transfer printing. After 20 min, the P_TP_ was released and the stamps were separated from the perovskite surface (Figure 3d), resulting in PSCs with CuI HTL (Figure 3e). Finally, gold top contacts were deposited on the transfer-printed CuI HTL (Figure 3f). A laboratory-fabricated device with transfer printed CuI and its cross-sectional image showing various layers are presented in Figure 3g.

**Figure 4:**
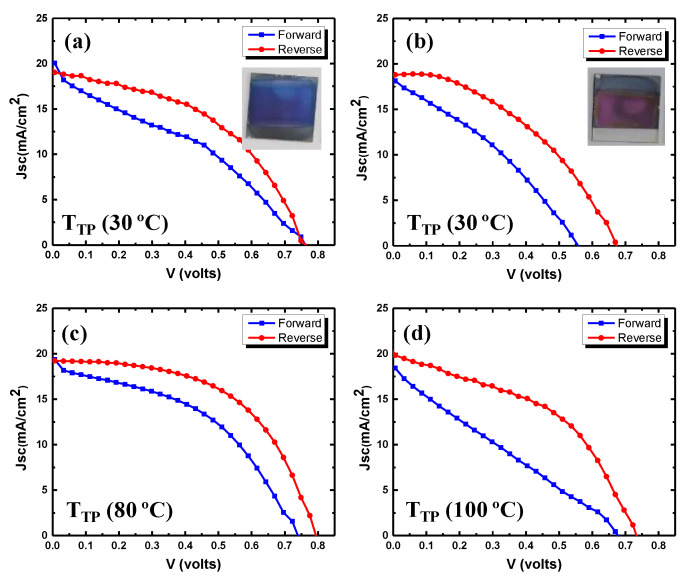
J–V curves for CuI HTL-based devices fabricated with iodization of (**a**) 20 and (**b**) 30 nm copper thin copper films (inset shows the laboratory-fabricated before-gold contact deposition). (**c**,**d**) devices fabricated using the iodization of 20 nm thin copper at transfer-printing pressure of 4 bar for 20 min at different transfer-printing temperatures, (**c**) 80 and (**d**) 100 °C.

The current density–voltage (J–V) curves of the CuI-based champion devices fabricated with 20 and 30 nm thickness of copper (iodized at 10 °C), measured under the standard AM 1.5 G illumination condition, are shown in Figure 4a,b, and corresponding photovoltaic parameters are listed in Table 1. A PSC with 20 nm thickness of copper yields a PCE of 6.6%, while devices with 30 nm thickness result in a decreased efficiency of 5.3%. The devices with 20 nm thickness of copper showed an open-circuit voltage (V_OC_) of 764 mV, a short-circuit current density (J_SC_) of 19.0 mA cm^−2^ and a fill factor of (FF) of 45.4%. These results reveal that a suitable thickness of CuI HTL is favorable for the better photovoltaic performance of PSCs [41]. Considering the point, devices with 20 nm thickness of copper film were investigated for further study to improve the photovoltaic performance of CuI-based PSCs.

**Figure 5:**
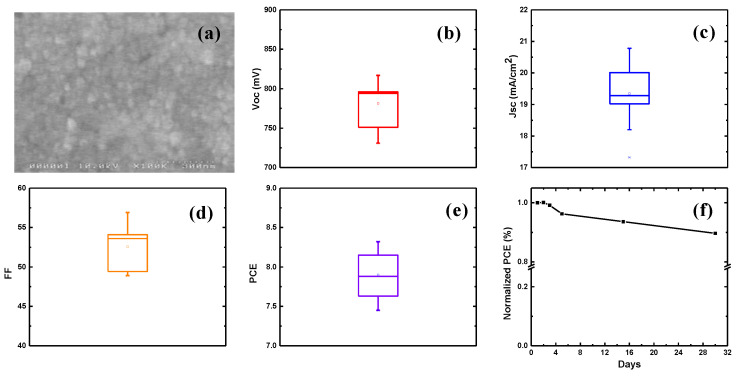
(**a**) Morphology of the CuI film (obtained from the iodization of 20 nm thin copper films at 10 °C) after transfer-printing on perovskite surface at 80 °C for 20 min at 4 bar pressure. (**b**–**e**) Whisker plots showing the statistical distribution of (**b**) V_OC_, (**c**) J_SC_, (**d**) FF and (**e**) PCE, based on 10 devices with transfer-printed CuI films, (**f**) Time–PCE curve for the champion device obtained with transfer-printed CuI HTL.

Further, the transfer-printing temperature (T_TP_) was optimized, keeping the thickness of copper film (20 nm) and iodization conditions constant. The PSCs were fabricated at T_TP_ of 30, 80, 100 and 150 °C. The devices fabricated at 80 °C T_TP_ achieved an improved PCE of 8.3%, with an open-circuit voltage of 795 mV, a short-circuit current of 19.3 mA/cm^−2^ and a fill factor of 53.8% (Figure 4c). This improved performance can be attributed to better interfacial properties due to increased T_TP_ promoting the charge transfer. To confirm the charge, transfer capabilities at the perovskite/(CuI) interface in these devices, photoluminescence (PL) quenching of the perovskite emission and dark J–V characteristics were examined (Figure S3, Supplementary Materials). From the PL spectra (Figure S3a), it can be seen that pristine perovskite films produced a strong fluorescence signal, which evidently decreased after the CuI HTL deposition. Both the devices, fabricated at T_TP_ of 30 and 80 °C, significantly quenched the perovskite emission signal, but a relatively greater PL quenching in the samples obtained at 80 °C, clearly indicates an improved hole extraction in these devices [25]. Further the dark current voltage (J–V) characteristics (Figure S3b) showed a lower leakage current, suggesting an increased shunt resistance in the devices obtained at higher (80 °C) T_TP_, which is also reflected in the increased FF of these devices [48,49]. The devices fabricated at T_TP_ of 100 °C showed a decreased PCE of 6.6% with a short-circuit current density of 19.9 mA/cm^−2^ and a fill factor of 45.2%. In order to confirm the effect of T_TP_ on the device performance, devices with higher transfer-printing temperature (150 °C) were fabricated, and the results showed a very poor power-conversion efficiency of 2.8% (Figure S4 and Table S2). These results confirm that increasing the T_TP_ initially improves the performance of devices but, beyond a certain temperature, device performance degrades, which may be due to the degradation of the CuI/perovskite interface or the perovskite itself. Moreover, PDMS has a volumetric coefficient of thermal expansion of 9.6 × 10^−4^ °C^−1^, and it can undergo volume expansion and swelling, during high-temperature transfer-printing [50]. It can induce mechanical stress and/or mechanical failures such as wrinkles and cracks on CuI, resulting in process defects and leading to the poor performance of the devices. The morphology of transfer-printed CuI layers (T_Iod_ = 10 °C, with 20 nm Cu) in champion devices fabricated at T_TP_ of 80 °C for 20 min at 4 bar pressure is shown in Figure 5a. After transfer-printing, the copper iodide is very compact and uniform. The top-view SEM morphology of the complete device after metal (gold) contact evaporation is shown in the Supplementary Materials (Figure S5).

The reproducibility of the CuI HTL device performance and the statistical distribution of the four main parameters of the PSCs (Voc, J_SC_, FF, and PCE) for ten devices (fabricated with conditions, t_Cu_ = 20 nm, T_Iod_ = 10 °C, T_TP_ = 80 °C, t_TP_ = 20 min and P_TP_ = 4 bar), in the form of whisker plots, are shown in Figure 5b–e. From the whisker plots, it can be seen that CuI-based devices showed an average PCE of 7.9 ± 0.4%. Further, the stability of CuI HTL-based devices was studied over a period of one month by storing the devices inside a desiccator with a vacuum. The time–PCE curve for the champion device is shown in Figure 5f. From the figure, it can be seen that the CuI HTL devices are quite stable, retaining ~90% of the initial power-conversion efficiency after a period of 30 days.

**Figure 6:**
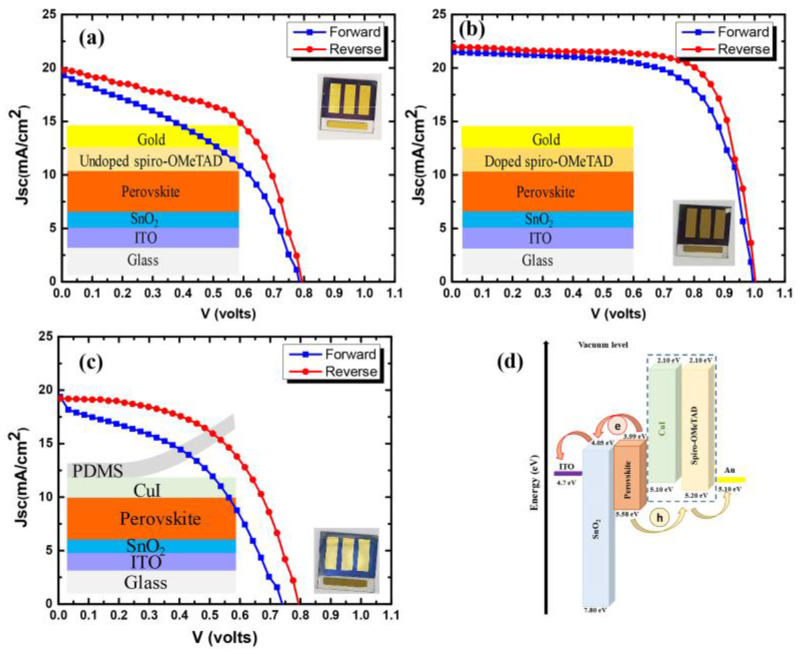
J–V curves for devices fabricated with different HTLs: (**a**) undoped spiro-OMeTAD, (**b**) doped spiro-OMeTAD, (**c**) transfer-printed CuI, (**d**) proposed charge transfer in the fabricated devices.

Spiro-OMeTAD is the most widely used HTL in the n–i–p-structured perovskite solar cells. Therefore, in order to compare the performance of our CuI HTL-based devices with the spiro-OMeTAD, the J–V characteristics of the champion devices made with spiro-OMeTAD (doped and undoped) and CuI HTL were analyzed. The obtained J–V curves are shown in Figure 6a–c, and the corresponding photovoltaic parameters for the champion devices are summarized in Table 2. It is undoubtedly clear from the table that the photovoltaic performance of the devices made with doped spiro-OMeTAD HTL is outstanding, achieving a PCE of 16.1% with a high open-circuit voltage of 1001 mV, a short-circuit current density of 21.6 mA/cm^−2^ and a fill factor of 73.2%. However, from the table, it can be seen that the devices made with updoped spiro-OMeTAD have comparable efficiencies with those made with the CuI HTL. The updoped spiro-OMeTAD HTL devices showed an open-circuit voltage of 796 mV, a short-circuit current of 19.9 mA/cm^−2^ and a fill factor of 55.5%, resulting in a PCE of 8.8%, comparable to CuI HTL-based devices. The charge-transfer scheme in the spiro-OMeTAD and CuI HTL-based devices is shown in Figure 6d. From the figure, it can be seen that there is a larger energy gap of 0.48 eV between the perovskite and CuI as compared to 0.38 eV for perovskite and spiro-OMeTAD. This larger energy difference in the CuI HTL-based devices is affecting the charge transfer, resulting in a lower PCE for CuI HTL-based PSCs [42]. Furthermore, to check the superiority of our CuI HTL fabrication method, the device was made with direct copper evaporation on the perovskite layer and iodization (under the same iodization conditions as for champion devices with transfer-printing). The schematic of device fabrication, the corresponding J–V curve and photovoltaic parameters are presented in Figure S6 and Table S3, respectively. The fabricated devices showed a very small short-circuit current, resulting in low efficiency.

A comparison of CuI HTL-based devices, both those fabricated in the present study and those previously fabricated with other techniques and structures, is presented in Table 3. As listed in Table 3, all of the previous fabricated devices are based on the TiO_2_ ETL, which requires high-temperature processing and imposes certain limits on the processing and choice of substrates. In the present study, the devices were fabricated with low-temperature-processed SnO_2_ ETL, to lower the processing temperature, allowing a variety of substrates for processing. Furthermore, the higher PCE of the transfer-printed CuI HTL devices in the present study suggests the feasibility of the low-temperature fabrication of CuI HTL-based efficient devices. The combination of optimized copper thickness, iodization temperature, transfer-printing temperature, pressure and time has resulted in improved PCE in the present study.

Finally, a cost-efficiency comparison for CuI- and spiro-OMeTAD-based devices has been estimated and is presented in Figure S7 (Supplementary Materials). For a better estimation, we have selected the highest efficiency (25.6%) of the spiro-OMeTAD-based devices from the literature, while for the CuI-based devices, we have selected the PCE obtained in the present study [12]. From the plots, it can be seen that spiro-OMeTAD has approximately 100-times higher cost for one gram of the material. More specifically, in term of cost and efficiency, spiro-OMeTAD-based devices have approximately 33 times higher cost than that of CuI for every 1% of the efficiency. These estimations clearly suggest the utility of CuI in the perovskite solar cells.

A comparison of present HTM with other dopant-free HTMs for planar SnO_2_-ETL based (n–i–p) PSCs in terms of PCE, mobility, cost, stability and ease of HTM synthesis is presented in Table S1. From the table, it can be seen that the PCE of the CuI HTM-based devices needs to be further improved. Still, the material is the least-expensive, has a very high mobility, good stability and can be synthesized in ambient environment with a very simple and less time-consuming method. All of these characteristics suggest the practical feasibility of the material as an HTM in the PSCs. Future studies focusing on the charge-transfer scheme can result in improved PCE for CuI HTL-based perovskite solar cells and make them potential candidates for substituting for the doped spiro-OMeTAD.

## 4. Conclusions

In short, in this study, we have investigated the ambient atmosphere fabrication of perovskite solar cells with a low-cost CuI material with a transfer-printing method, utilizing a low-temperature-processed electron transport layer (SnO_2_). A variety of ITO/SnO_2_/perovskite/HTL(spiro-OMeTAD and CuI)/Au devices were fabricated, and champion devices were compared. Fully ambient-atmosphere-processed perovskite devices with transfer-printed CuI showed a promising efficiency of 8.3%. Indeed, the performance of transfer-printed CuI devices is lower than doped spiro-OMeTAD-based PSCs, but fabricated devices still have comparable efficiency as undoped spiro-OMeTAD. This clearly suggests the possibility of replacing highly expensive and undoped spiro-OMeTAD with transfer-printed CuI HTL.

## Figures and Tables

**Table 1:** Device parameters for CuI HTL-based devices fabricated with different thicknesses of copper films (t_Cu_) and transfer-printing temperatures (T_TP_).

Cu Film Thickness (nm)	T_TP_ (°C)	Scan Direction	V_oc_ (mV)	J_sc_ (mA/cm^2^)	FF (%)	PCE (%)	H.I.
30	30	Forward Reverse	557 669	18.1 18.8	32.9 42.1	3.3 5.3	0.37
20	30	Forward Reverse	758 764	19.3 19.0	35.9 45.4	5.3 6.6	0.20
20	80	Forward Reverse	742 795	18.8 19.3	44.1 53.8	6.3 8.3	0.24
20	100	Forward Reverse	675 728	18.4 19.9	25.3 45.2	3.2 6.6	0.31

**Table 2:** Device parameters for devices fabricated with different HTL materials.

HTL Material	Scan Direction	V_oc_ (mV)	J_sc_ (mA/cm^2^)	FF (%)	PCE (%)	H.I.
Undoped spiro-OMeTAD	Forward Reverse	779 796	19.5 19.9	42.6 55.5	6.5 8.8	0.26
Doped spiro-OMeTAD	Forward Reverse	994 1001	21.4 21.6	67.7 73.2	14.5 16.1	0.09
Transfer-printed CuI	Forward Reverse	742 795	18.8 19.3	44.1 53.8	6.3 8.3	0.24

**Table 3:** Comparison of (n–i–p) device structures and parameters for CuI HTL-based devices.

S. No.	Device Structure	CuI Deposition Method	V_OC_ (V)	J_SC_ (mA/cm^2^)	FF (%)	PCE (%)	Ref.
**1.**	TiO_2_/CH_3_NH_3_PbI_3_/CuI	Solution pumping	0.55	17.8	0.62	6.0	[32]
**2.**	TiO_2_/CH_3_NH_3_PbI_3_/CuI_3−x_/CuI	Spray coating	0.61	22.3	0.42	5.8	[39]
**3.**	TiO_2_/CH_3_NH_3_PbI_3_/CuI	Doctor blade	0.78	16.7	0.57	7.5	[43]
**4.**	TiO_2_/CH_3_NH_3_PbI_3_/CuI	Gas–solid treatment	0.73	32.7	0.31	7.4	[51]
**5.**	TiO_2_/CH_3_NH_3_PbI_3_/CuI/Cu	Thermal evaporation	0.85	23.0	0.47	9.2	[40]
**6.**	TiO_2_/CH_3_NH_3_PbI_3_/CuI	Spin coating	0.42	14.7	0.40	2.2	[38]
**7.**	TiO_2_/CH_3_NH_3_PbI_3_/CuI_3−x_/CuI	Powder pressing	0.67	24.2	0.50	8.1	[42]
**8.**	TiO_2_/PVSK/CuI	Thermal evaporation	0.83	15.6	0.62	8.1	[41]
**9.**	SnO_2_/PVSK/CuI	Transfer printing	0.79	19.3	0.54	8.3	Present

## Data Availability

Not applicable.

## Supplementary Materials

The following supporting information can be downloaded at: https://www.mdpi.com/article/10.3390/nano12091467/s1, Figure S1: Schematic of iodization of Cu film. Figure S2: XRD and SEM of perovskite films. Figure S3: Steady-state photoluminescence (PL) curves for pristine perovskite film, perovskite with CuI HTLs deposited at different transfer-printing temperatures and a dark J–V curve for the devices fabricated with CuI HTLs deposited at different transfer-printing temperatures. Figure S4: J–V of CuI HTL device transfer-printed at 150 °C. Figure S5: Morphology of the solar cell device. Figure S6: Schematic of Cu iodization deposited directly on perovskite layer and comparison of solar cell performance. Figure S7: Cost efficiency comparison. Table S1: Comparison of CuI with other dopant-free HTMs for planar SnO_2_-ETL based (n–i–p) PSCs. Table S2: J–V parameter of CuI HTL device transfer-printed at 150 °C. Table S3: Device parameter comparison fabricated with different methods [19,20,21,22,23,24,25,26,27,28,29,30].

## Abbreviations

PSCs	*Perovskite solar cells*
PCEs	*Power-conversion efficiencies*
HTL	*Hole transport layer*
ETL	*Electron transport layer*
HTM	*Hole transport material*
CuI	*Cuprous iodide*
V_OC_	*Open-circuit voltage*
J_SC_	*Short-circuit current density*
FF	*Fill factor*
H.I.	*Hysteresis index*
ITO	*Indium tin oxide*
Li-TFSI	*Lithium bis- (trifluoromethylsulfonyl)imide*
4-tBP	*4-tert-butylpyridine*
SnO_2_	*Tin (IV) oxide*
Spiro-OMeTAD	*2,2′,7,7′-Tetrakis[N,N-di(4-methoxyphenyl)amino]-9,9′-spirobifluorene*
PDMS	*Polydimethylsiloxane*
t_Cu_	*Thickness of copper film*
T_Iod_	*Iodization temperature*
t_TP_	*Transfer-printing time*
P_TP_	*Transfer-printing pressure*
T_TP_	*Transfer-printing temperature*
J-V	*Current density–voltage*
XRD	*X-ray diffraction*
SEM	*Scanning electron microscope*
PL	*Photoluminescence*
