# Supplementary material for: Transfer-Printed Cuprous Iodide (CuI) Hole Transporting Layer for Low Temperature Processed Perovskite Solar Cells
Source: Nanomaterials (Basel). 2022 Apr 26;12(9):1467. doi: 10.3390/nano12091467 (PMC9101613; doi:10.3390/nano12091467)
Supplement: Supplementary file 1 [file nanomaterials-12-01467-s001.zip › nanomaterials-1693315-supplementary.pdf]

nanomaterials

Supplementary Information

Transfer-Printed Cuprous Iodide (CuI) Hole Transporting Layer for Low Temperature Processed Perovskite Solar Cells

Ravi P. Srivastava, Hyun-Suh Jung and Dahl-Young Khang *

Department of Materials Science and Engineering, Yonsei University, Seoul 03722, Korea; ravimme.iitk@gmail.com (R.P.S.); li5245@naver.com (H.-S.J.)
* Correspondence: dykhang@yonsei.ac.kr

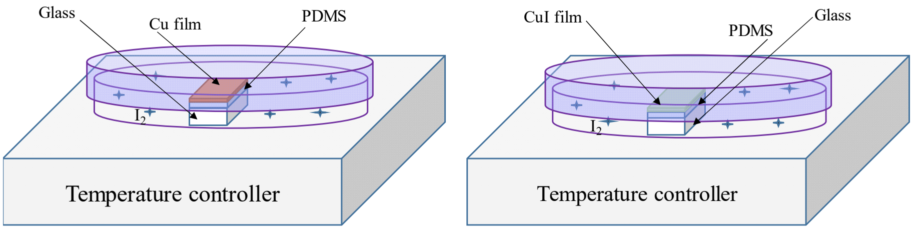

Figure S1. Schematic of iodization of copper films deposited on the glass/PDMS (**a**) before iodization (**b**) after iodization.

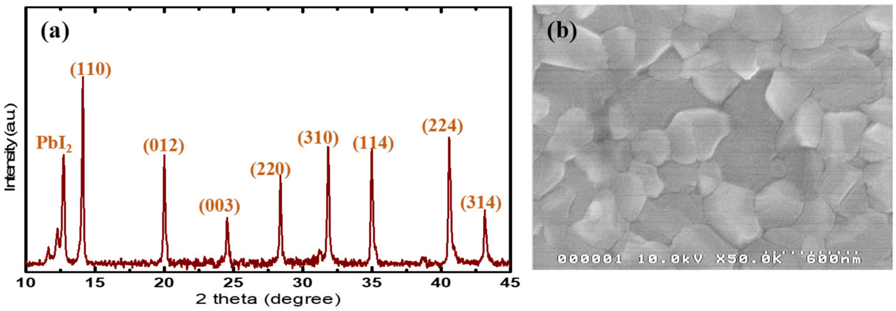

Figure S2. (**a**) XRD and (**b**) morphology of perovskite film.

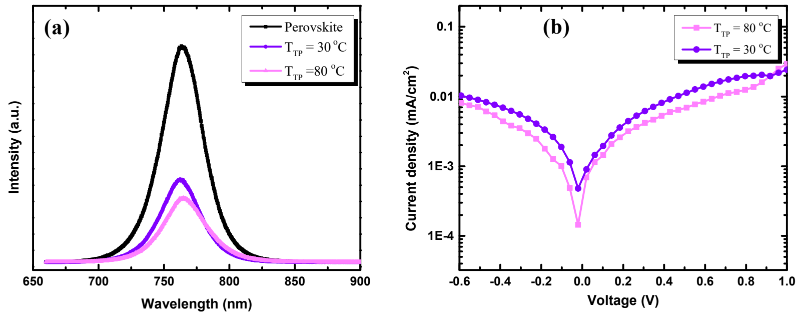

Figure S3. (**a**) Steady state photoluminescence (PL) curves for the pristine perovskite film and perovskite with CuI HTLs deposited at different transfer printing temperatures. (**b**) Dark J-V curve for the devices fabricated with CuI HTLs deposited at different transfer printing temperatures.

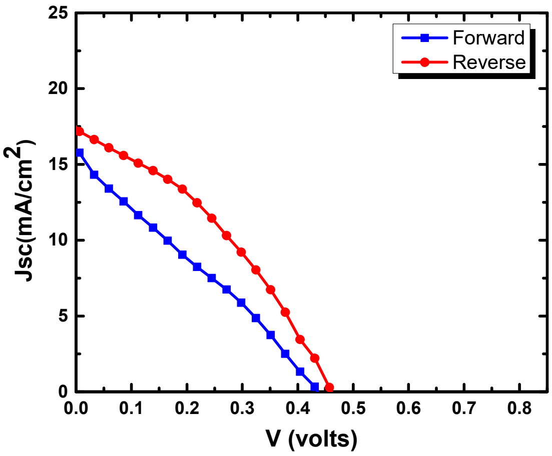

Figure S4. J-V characteristic of the CuI HTL based devices fabricated at 150 °C transfer printing temperature.

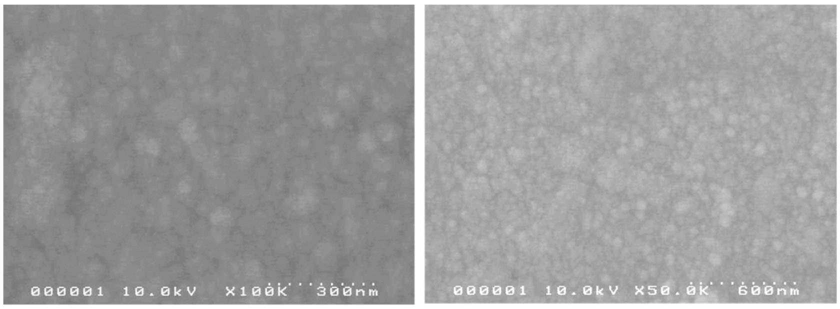

Figure S5. Morphology of glass/ITO/SnO_2/Perovskite/CuI/Au.

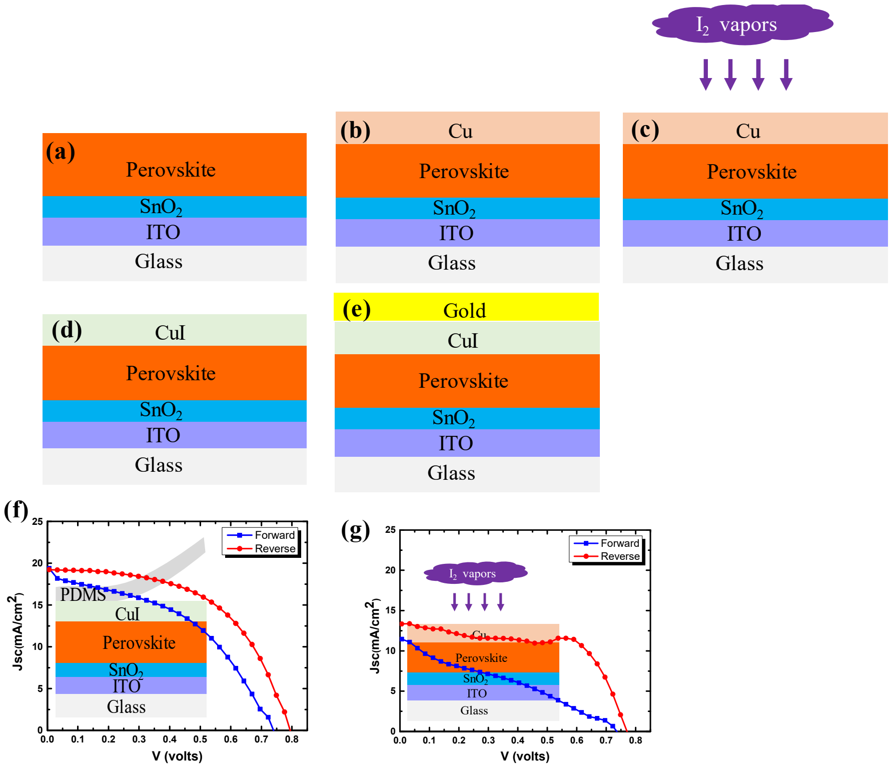

Figure S6. (a-e) Schematic of CuI HTL device fabrication using iodization of copper directly deposited on the perovskite layer. J-V curves for CuI HTL based devices fabricated with **(f)** transfer printing and **(g)** iodization of Cu directly deposited on the perovskite layer.

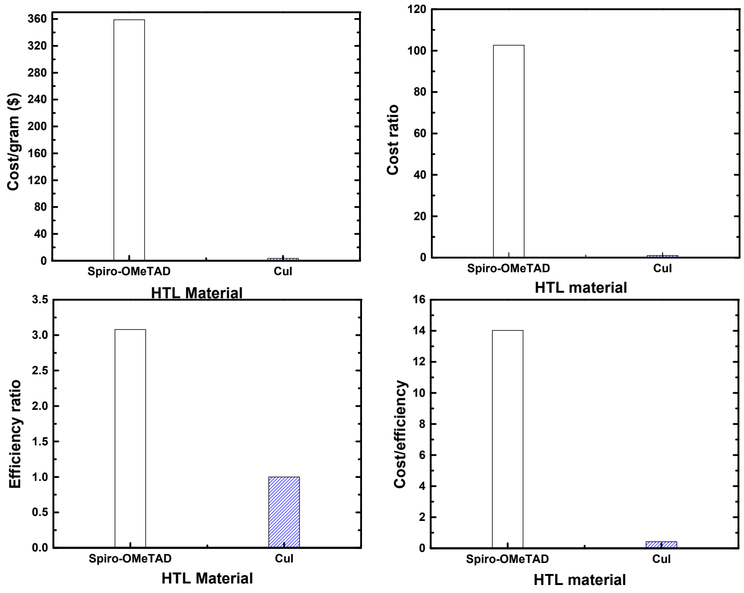

Figure S7. Cost efficiency comparison for Spiro-OMeTAD and CuI HTL based perovskite solar cells.

Table S1. Comparison of CuI with other dopant free HTMs for planar SnO_2-ETL based (n-i-p) PSCs.

S.No.	HTM	Mobility ($cm^2V^{-1}s^{-1}$)	PCE (%)	Stability		Material cost ($/g)	HTM synthesis	Ref. No.
				Aging time (h)	Retained PCE (%)			
1	M129	$4.23 X 10^{-4}$	19.5	800	94	28.29	Comlicated	[19]
2	TTE2	$6.18 X 10^{-4}$	20.0	1000	85	–	Comlicated	[20]
3	DTPC8-ThDTPA	$6.50 X 10^{-4}$	19.4	–	–	–	Complicated	[21]
4	DTPC13-ThTPA	$2.16 X 10^{-4}$	20.4	–	–	14.36	Complicated	[22]
5	IDTT-TPA	$6.46 X 10^{-4}$	15.7	–	–	–	Comlicated	[23]
6	HTB-OMe	$5.48 X 10^{-4}$	17.3	720	>75	–	Complicated	[24]
7	IDTC6-TPA	$4.26 X 10^{-4}$	15.4	160	94	–	Complicated	[25]
8	BTF4	$1.17 X 10^{-4}$	18.0	720	50	62.78	Complicated	[26]
9	HTM3	$2.10 X 10^{-5}$	14.2	700	90	9.73	Complicated	[27]
10	CuBuPc	$12.6 X 10^{-4}$	15.0	240	90	–	Comlicated	[28]
11	P2	$1.32 X 10^{-4}$	18.5	720	80	8.86	Moderate	[29]
12	PTEG	$1.64 X 10^{-3}$	19.8	200	>90	–	Complicated	[30]
13	CuI	1.73	8.3	720	90	3.51	Simple	Present work

* Simple (1-2 steps); Moderate (3-4 steps); Complicated (> 4 steps)

Table S2. J-V parameters for the CuI HTL based devices fabricated at 150 °C transfer printing temperature.

Cu Film Thickness (nm)	T_{TP} (°C)	Scan Direction	V_{oc} (mV)	J_{sc} (mA/cm^2)	FF (%)	PCE (%)	H.I.
20	150	Forward	431	15.70	27.17	1.81	0.36
		Reverse	460	17.49	34.58	2.81	

Table S3. Device parameters for CuI HTL based devices fabricated with different method.

Method	Scan Direction	V_{oc} (mV)	J_{sc} (mA/cm^2)	FF (%)	PCE (%)	H.I.
Direct copper deposition on perovskite and iodization	Forward	770	13.3	65.2	6.7	0.63
	Reverse	726	11.3	29.9	2.5	
Transfer printing of CuI	Forward	742	18.8	44.1	6.3	0.24
	Reverse	795	19.3	53.8	8.3	
